# COVID-19–Relevant Insights Into the Elevated Risk of Accidental Injuries in Survivors of SARS and Their Relatives in Taiwan: Retrospective Cohort Study

**DOI:** 10.2196/70608

**Published:** 2025-07-08

**Authors:** Chieh Sung, Chi-Hsiang Chung, Chien-An Sun, Chang-Huei Tsao, Daphne Yih Ng, Tsu-Hsuan Weng, Li-Yun Fann, Fu-Huang Lin, Wu-Chien Chien

**Affiliations:** 1 Department of Senior Citizen Care and Welfare, Deh Yu College of Nursing and Health, Keelung City, Taiwan; 2 Department of Medical Research, Tri-Service General Hospital, National Defense Medical Center, No. 325, Section 2, Chenggong Road, Taipei, Taiwan; 3 School of Public Health, National Defense Medical Center, Taipei, Taiwan; 4 Department of Public Health, College of Medicine, Fu-Jen Catholic University, New Taipei City, Taiwan; 5 Big Data Research Center, College of Medicine, Fu-Jen Catholic University, New Taipei City, Taiwan; 6 Department of Microbiology & Immunology, National Defense Medical Center, National Defense Medical Center, Taipei, Taiwan; 7 Department of Family Medicine, Tri-Service General Hospital, National Defense Medical Center, Taipei, Taiwan; 8 Graduate Institute of Medical Sciences, National Defense Medical Center, Taipei, Taiwan; 9 Department of Nursing, Taipei City Hospital, 145 Zhengzhou Road, Datong District, Taipei, 103212, Taiwan, 886 0225553000; 10 Department of Nursing, Tzu Chi University, Hualien, Taiwan

**Keywords:** SARS, accidental injuries, COVID-19 pandemic, psychosocial impacts, intentional injuries, survivors of SARS, accidental falls, poisoning, psychological stress, caregivers, population surveillance, severe acute respiratory syndrome

## Abstract

**Background:**

The 2003 outbreak of severe acute respiratory syndrome (SARS), caused by a novel coronavirus, heavily impacted Taiwan’s health care system, triggering clinical crises and lasting effects among affected individuals and families. The first case in Taiwan was identified on February 25, 2003, and the final case was reported on June 15, 2003. During the epidemic, 346 people were diagnosed with SARS, leading to 37 deaths. Outbreaks also occurred in China, Singapore, and Toronto (Canada), showing the vulnerability of global health systems to new zoonotic diseases. Clinically, SARS causes high fever and severe lung inflammation. Survivors often had long-term lung problems, including fibrosis, and bone issues like osteonecrosis, mostly due to high-dose steroid treatment. Although studies have looked at long-term outcomes—especially lung and bone issues—none followed patients beyond 7 years. The COVID-19 pandemic further revealed gaps in understanding how serious viral infections affect wider health areas, including unintentional and intentional injuries. Data on related hospitalizations also remain limited.

**Objective:**

This study aimed to investigate the long-term risk of both unintentional and intentional injuries among survivors of SARS and their relatives, using a nationwide population-based cohort.

**Methods:**

This retrospective cohort study used data from Taiwan’s National Health Insurance Research Database, focused on 285 individuals diagnosed with SARS in 2003 and 699 of their relatives, matched in a 1:10 ratio with controls. Injury risks were assessed using Fine and Gray’s competing risk models, adjusting for sociodemographic and clinical covariates, over a follow-up period of up to 15 years.

**Results:**

Survivors of SARS exhibited a significant increase in the risk of accidental injuries, with an adjusted hazard ratio (AHR) of 1.631 (95% CI 1.184-2.011; *P*<.001), indicating persistent physiological vulnerabilities postinfection. Family members of survivors of SARS also had elevated injury risk (AHR 1.572, 95% CI 1.148-1.927; *P*<.001), possibly due to stress and caregiving burdens. Subgroup analysis showed increased risks for poisoning (AHR 2.701, 95% CI 1.956-4.084; *P*<.001) and falls (AHR 1.524, 95% CI 1.102-1.878; *P*=.003) among survivors. Relatives faced higher risks for traffic incidents (AHR 2.003, 95% CI 1.462-2.459), poisoning (AHR 1.531, 95% CI 1.120-1.886), medical incidents, falls (AHR 1.802, 95% CI 1.324-2.214), and crushing injuries (AHR 2.469, 95% CI 1.803-3.026; all *P*<.001). These findings highlight the need for targeted preventive measures to address long-term health risks in both survivors of SARS and their families.

**Conclusions:**

Survivors of SARS and their relatives face increased injury risks, highlighting long-term physical and psychosocial vulnerabilities after severe infectious outbreaks. These findings suggest that health care systems should provide preventive and supportive measures to mitigate long-term impacts for those affected by pandemics.

## Introduction

An outbreak of severe acute respiratory syndrome (SARS) caused by a novel coronavirus severely affected Taiwan in 2003 [1], with the first confirmed diagnosis of SARS on February 25, 2003, and the last diagnosis on June 15, 2003. Through this endemic outbreak, there were 346 patients diagnosed with SARS, and 37 died among these patients [2], and there were also SARS outbreaks in China, Singapore, and Toronto, Canada, at about the same time [2]. The COVID-19 pandemic has had a global impact on all facets of medicine, resulting in significant stress and challenges in the medical community.

The main clinical manifestations after infection were high fever and severe lung inflammation [3,4]. The patients who survived had residual pulmonary fibrosis, as well as osteonecrosis, resulting from treatment with large doses of steroid pulse therapy [5]. Several studies have investigated the long-term outcomes of recovered patients with SARS, especially with respect to lung and bone damage, but no follow-up periods of more than 7 years have been reported [6,7]. Furthermore, there is a scarcity of research regarding the effects of COVID-19 on unintentional injuries and intentional injuries, as well as admission rates [8]. Survivors of respiratory infections like SARS and COVID-19 frequently report persistent symptoms that severely impact their daily functioning and quality of life. These symptoms include chronic fatigue, muscle weakness, and neurological manifestations such as “brain fog” and cognitive deficits, which are well-documented consequences of both diseases [9-11]. Beyond the physical impairments, there is growing evidence highlighting the long-term mental health burden of these infections, with elevated rates of anxiety, depression, posttraumatic stress disorder, and sleep disturbances reported to persist for months or even years after recovery [12-14]. These multifaceted impairments may heighten the risk of accidental injuries in affected individuals. Specifically, cognitive dysfunction, such as reduced attention, slower processing speed, and impaired judgment, is known to increase vulnerability to various types of accidents, including falls and motor vehicle incidents [15,16]. Moreover, physical limitations like chronic fatigue and muscle weakness directly compromise balance, coordination, and reaction time, thus elevating the likelihood of falls, particularly in older adults [17,18]. The increased prevalence of mental health disorders postpandemic may also lead to greater reliance on psychotropic medications, which carry their own risks, including sedation and impaired gait, further contributing to the elevated risk of falls [19]. While the direct link between SARS and subsequent accidental injuries has been underexplored, the parallels in the long-term physical and psychological consequences observed in survivors of COVID-19 strongly suggest that survivors of SARS may similarly face increased vulnerability to injuries [20,21].

The implementation of social distancing and quarantine measures during COVID-19 likely led to a decrease in trauma surgery cases, as restrictions reduced high-risk activities [8]. We hypothesized that SARS is associated with an increased risk of these injuries and tested this hypothesis through a nationwide cohort study using the National Health Insurance Research Database (NHIRD), which includes claims data for Taiwan’s entire population [22].

## Methods

### Data Sources

Established in 1995, Taiwan’s National Health Insurance (NHI) Program is a mandatory and universal health insurance system that covers approximately 23 million beneficiaries—over 99% of the population [22]. The NHIRD contains comprehensive and detailed data regarding the total outpatients and inpatients [23-33].

We selected an inpatient dataset from 2000 to 2015 from the NHIRD, with individual diagnoses coded using the *International Classification of Diseases, Ninth Revision, Clinical Modification* (*ICD-9-CM*). The details of the NHI program have been documented in several previous studies [34-39]. Several studies have also confirmed the precision and validity of these diagnoses [40-42].

### Ethical Considerations

This study was conducted in accordance with the Code of Ethics of the World Medical Association (Declaration of Helsinki). The Institutional Review Board of the Tri-Service General Hospital approved this study (IRB: TSGHIRB No. E202416040) on November 4, 2024, and waived the need for individual consent since all identification data were encrypted in the NHIRD. The study was granted review exemption status, and no artificial intelligence tools were used in data analysis or study preparation. As this was a secondary data analysis of existing administrative data, no direct patient recruitment or contact was performed. The database provides a representative, real-world population sample, allowing for longitudinal follow-up and comprehensive capture of medical events without selection bias inherent in traditional recruitment. No generative artificial intelligence tools were used in any part of the study writing process.

### Study Design and Sampled Participants

This retrospective matched-cohort study used inpatient data from January 1, 2000, to December 31, 2015. Each patient with SARS was required to have a diagnosis in an inpatient setting with *ICD-9-CM* codes 480.8 and 480.9. We selected 285 patients with SARS from 2003 and matched them with 2850 controls at a 1:10 ratio. Additionally, we identified 699 relatives of patients with SARS and matched them with 6990 controls, also at a 1:10 ratio. Propensity score matching was used using variables including gender, age, insurance premium, comorbidities, location, level of care, and index date. Exclusion criteria for the cohorts included individuals with unknown gender and those diagnosed with psychiatric disorders or pneumonia and influenza (*ICD-9-CM* codes 480‐488) before the index date. The index date was defined as the time when individuals were first diagnosed with SARS within the 1-year study period (Table S1 in Multimedia Appendix 1).

All SARS participants and their matched controls were followed from the index date until the occurrence of an injury, including unintentional injuries (traffic accidents, poisoning, medical-related incidents, falls, burns and fires, drowning, suffocation, crushing injuries, adverse drug reactions, and other unintentional injuries), intentional injuries (suicide, or homicide or abuse), or until death, withdrawal from the NHI program, or the end of 2015.

The covariates include sociodemographic characteristics and comorbidities. Sociodemographic characteristics included gender, age (18‐44, 45‐64, ≥65 y), education (<12 years; ≥12 y), monthly insured premiums, urbanization levels, regions of residence, and levels of medical care. The monthly insured premiums have been divided into three categories in New Taiwan Dollars: <18,000, 18,000‐34,999, and ≥35,000. The urbanization level was defined by population and certain indicators of the city’s level of development. Level 1 urbanization was defined as having a population greater than 1,250,000 people. Level 2 urbanization was defined as having a population between 500,000 and 1,250,000. Urbanization levels 3 and 4 were defined as having a population between 150,000 and 500,000 and less than 150,000, respectively [43]. The Charlson Comorbidity Index (CCI) is one of the most widely used comorbidity indices [44,45], which consists of 22 conditions [46]. We used the CCI to quantify the comorbidities since it could predict the in-hospital mortality or outcome in patients with severe adult respiratory infection and other infections [47-49].

### Statistical Analysis

Statistical analyses were conducted using SPSS (version 22; IBM Corp). Categorical variables were examined with the chi-square test, while continuous variables, expressed as means (SDs), were compared using 2-sample *t* tests. To evaluate the risk of injuries among patients with and without exposure to patients with SARS or their relatives, competing risk analysis was performed using Fine and Gray’s model to calculate adjusted hazard ratios (AHRs) and 95% CIs, adjusting for sociodemographic characteristics and comorbidities. Additionally, Cox proportional hazards regression and sensitivity analyses were used to assess injury risk through the end of 2015. The Kaplan-Meier method was used to compare the cumulative incidence of injuries between the exposed groups and control cohorts, with differences evaluated using the log-rank test. A *P* value of <.05 was considered statistically significant.

## Results

### Study Cohort Characteristics

**Figure 1.**
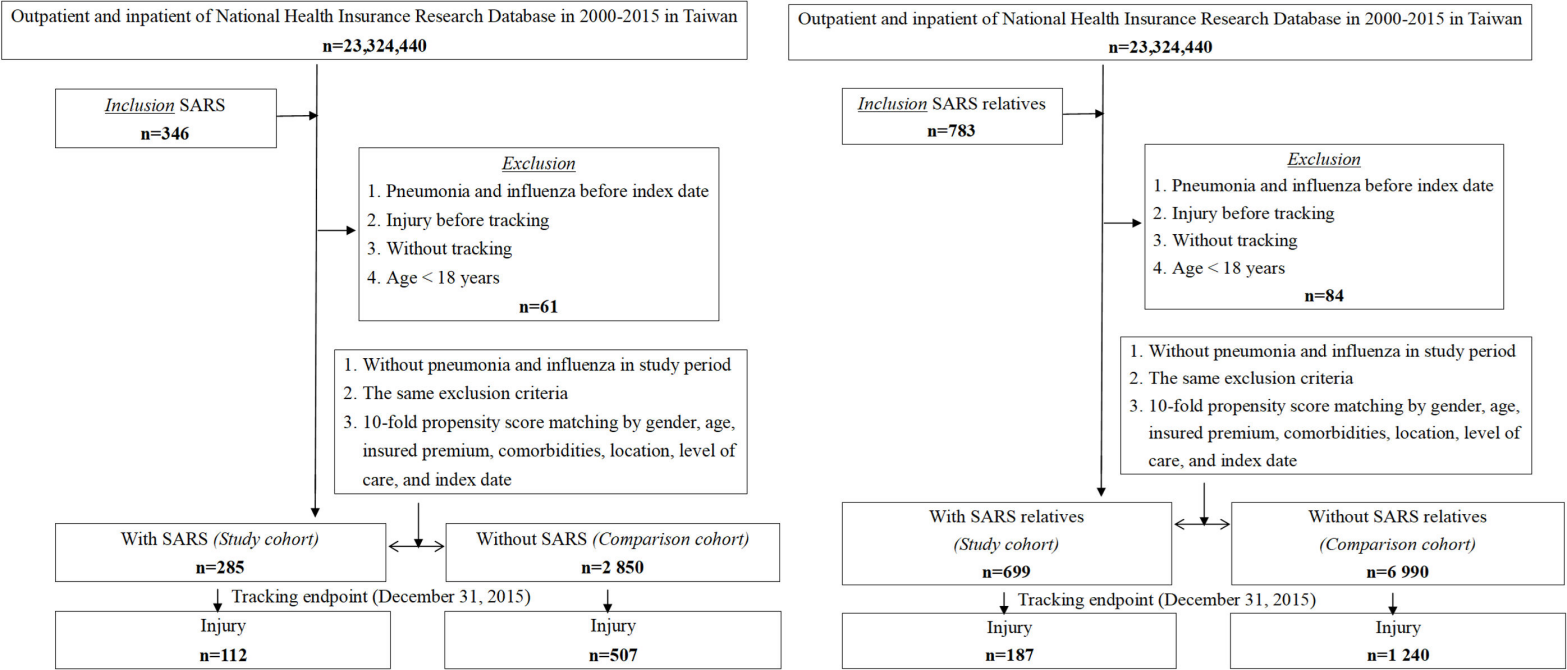
Flowchart detailing the selection of survivors of severe acute respiratory syndrome (SARS) and their first-degree relatives and matched control cohorts from the National Health Insurance Research Database in a nationwide retrospective cohort study conducted in Taiwan (2000‐2015).

Table 1 presents the demographic and clinical characteristics—including gender, age, education level, monthly insured premiums, urbanization level, region of residence, comorbidities, and levels of medical care—of patients with SARS and their relatives, both with and without SARS. Compared to the control group, there were no significant differences in these covariates between patients with SARS, their relatives, and the controls. The participant flowchart is presented in Figure 1.

**Table 1.** Baseline demographic and clinical characteristics of survivors of SARS, first-degree relatives, and matched controls in a nationwide retrospective cohort study (Taiwan, 2000‐2015)^a^.

	Patients with SARS							Relatives of patients with SARS						
	Total (n=3135)		With (n=285)		Without (n=2850)		*P* value^b^	Total (n=7689)		With (n=699)		Without (n=6990)		*P* value^b^
Gender, n (%)							≥.99							≥.99
Male	1166 (37.19)		106 (37.19)		1060 (37.19)			4422 (57.51)		402 (57.51)		4020 (57.51)		
Female	1969 (62.81)		179 (62.81)		1790 (62.81)			3267 (42.49)		297 (42.49)		2970 (42.49)		
Age (years), mean (SD)	49.67 (12.24)		49.12 (12.18)		49.73 (12.25)		.42	46.72 (12.20)		46.69 (12.15)		46.72 (12.21)		.95
Age groups (years), n (%)							≥.99							≥.99
18‐44	1562 (49.82)		142 (49.82)		1420 (49.82)			4411 (57.37)		401 (57.37)		4010 (57.37)		
45‐64	1012 (32.28)		92 (32.28)		920 (32.28)			2695 (35.05)		245 (35.05)		2450 (35.05)		
≥65	561 (17.89)		51 (17.89)		510 (17.89)			583 (7.58)		53 (7.58)		530 (7.58)		
Insured premium (New Taiwan $)^c^, n (%)							.48							.98
<18,000	3068 (97.86)		281 (98.60)		2787 (97.79)			7573 (98.49)		689 (98.57)		6884 (98.48)		
18,000‐34,999	48 (1.53)		2 (0.70)		46 (1.61)			59 (0.77)		5 (0.72)		54 (0.77)		
≥35,000	19 (0.61)		2 (0.70)		17 (0.60)			57 (0.74)		5 (0.72)		52 (0.74)		
Marital status, n (%)							.38							.92
Without	1465 (46.73)		126 (44.21)		1339 (46.98)			3556 (46.25)		322 (46.07)		3234 (46.27)		
With	1670 (53.27)		159 (55.79)		1511 (53.02)			4133 (53.75)		377 (53.93)		3756 (53.73)		
Education levels (years), n (%)							.95							≥.99
<12	1625 (51.83)		147 (51.58)		1478 (51.86)			3941 (51.26)		359 (51.36)		3582 (51.24)		
≧12	1510 (48.17)		138 (48.42)		1372 (48.14)			3748 (48.74)		340 (48.64)		3408 (48.76)		
CCI_R^d^, n (%)							≥.99							≥.99
0	1738 (55.44)		158 (55.44)		1580 (55.44)			5288 (68.77)		482 (68.96)		4806 (68.76)		
1	638 (20.35)		58 (20.35)		580 (20.35)			1471 (19.13)		131 (18.74)		1340 (19.17)		
≥2	759 (24.21)		69 (24.21)		690 (24.21)			930 (12.10)		86 (12.30)		844 (12.07)		
Location, n (%)							.93							≥.99
Northern Taiwan	1437 (45.84)		132 (46.32)		305 (45.79)			3579 (46.55)		325 (46.49)		3254 (46.55)		
Middle Taiwan	597 (19.04)		54 (18.95)		543 (19.05)			1464 (19.04)		133 (19.03)		1331 (19.04)		
Southern Taiwan	961 (30.65)		87 (30.53)		874 (30.67)			2352 (30.59)		214 (30.62)		2138 (30.59)		
Eastern Taiwan	135 (4.31)		11 (3.86)		124 (4.35)			284 (3.69)		26 (3.72)		258 (3.69)		
Outlying islands	5 (0.16)		1 (0.35)		4 (0.14)			10 (0.13)		1 (0.14)		9 (0.13)		
Urbanization level, n (%)							.95							≥.99
1 (the highest)	1651 (52.66)		154 (54.04)		1497 (52.53)			4154 (54.03)		378 (54.08)		3776 (54.02)		
2	779 (24.85)		69 (24.21)		710 (24.91)			1875 (24.39)		170 (24.32)		1705 (24.39)		
3	535 (17.07)		46 (16.14)		489 (17.16)			1245 (16.19)		113 (16.17)		1132 (16.19)		
4 (the lowest)	170 (5.42)		16 (5.61)		154 (5.40)			415 (5.40)		38 (5.44)		377 (5.39)		
Level of care, n (%)							.98							≥.99
Hospital center	1634 (52.12)		149 (52.28)		1485 (52.11)			4013 (52.19)		365 (52.22)		3648 (52.19)		
Regional hospital	1170 (37.32)		107 (37.54)		1063 (37.30)			2889 (37.57)		262 (37.48)		2627 (37.58)		
Local hospital	331 (10.56)		29 (10.18)		302 (10.60)			787 (10.24)		72 (10.30)		715 (10.23)		

a This table presents the demographic and clinical baseline characteristics of survivors of SARS (n=285), their first-degree relatives (n=699), and matched non-SARS controls (n=2850 and n=6990, respectively), extracted from the National Health Insurance Research Database in Taiwan. The data spans from 2000 to 2015 and includes variables such as gender, age, marital status, education level, comorbidity index, insurance premiums, and health care access. The groups were matched 1:10 based on age, gender, comorbidities, and other demographic factors. Statistical significance was assessed using 2-tailed chi-square or Fisher exact tests for categorical variables and 2-tailed *t* tests for continuous variables.

b *P* values*:* chi-square and Fisher exact test on category variables and *t* test on continue variables.

c NT$1=US $0.035.

d CCI: Charlson Comorbidity Index, Revised.

### Patients With SARS Kaplan-Meier Curves for the Cumulative Incidence of Injury

**Figure 2.**
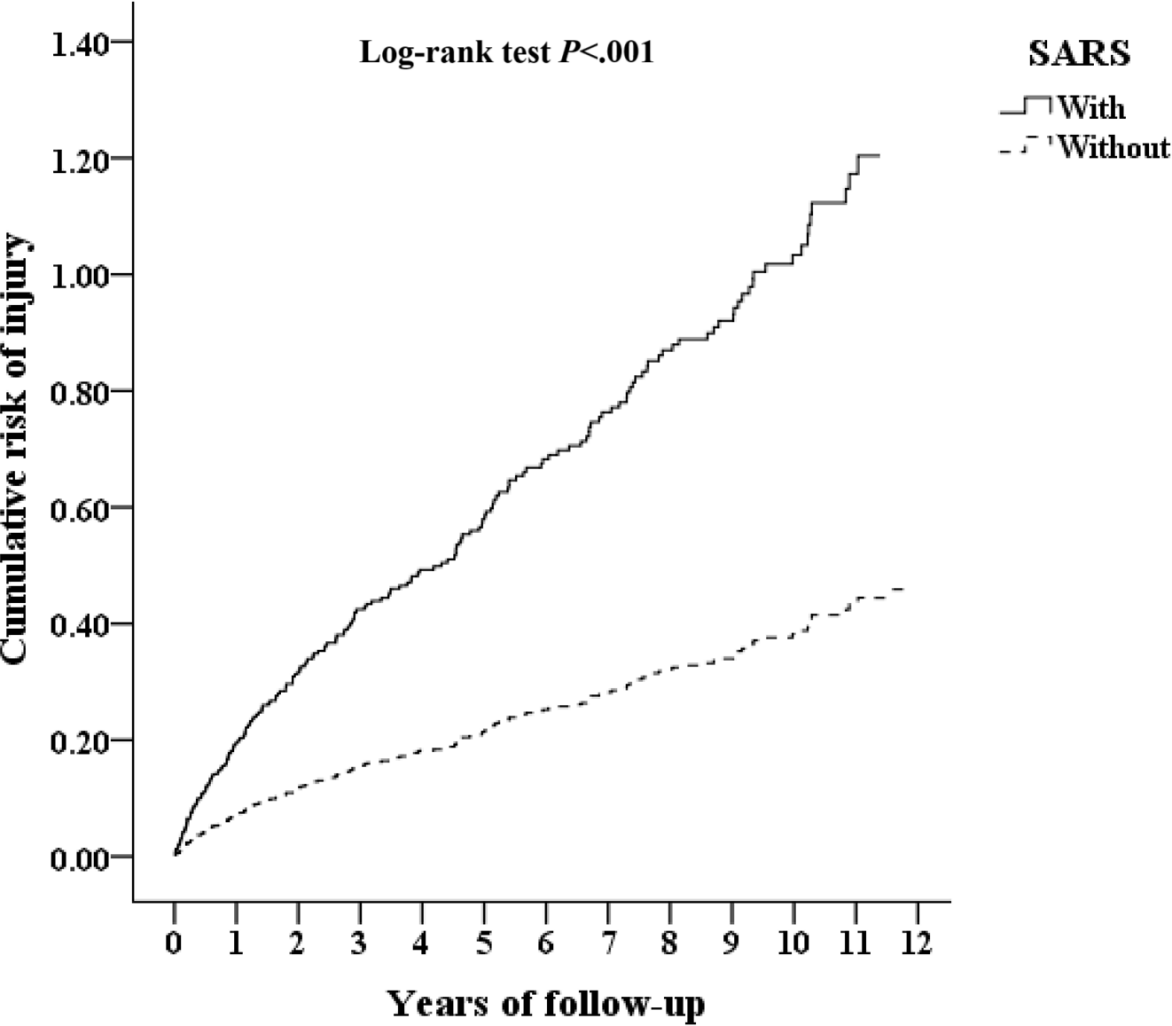
Kaplan-Meier survival curves illustrating the cumulative incidence of injury among the survivors of severe acute respiratory syndrome (SARS) and matched controls aged 18 years or older in a nationwide retrospective cohort study (Taiwan, 2000–2015). Statistical significance was determined using the log-rank test (*P*<.001).

In total, 285 patients were diagnosed with SARS during the study period. During the follow-up period, 112 in the SARS group (n=285) and 507 in the control group (n=2850) developed injury (5560.38 vs 2284.63 per 100,000 person-years). Figure 2 reveals that the difference between the two cohorts in the injury was significant (log-rank test; *P*<.001).

### Relatives of Patients With SARS Kaplan-Meier Curves for the Cumulative Incidence of Injury

**Figure 3.**
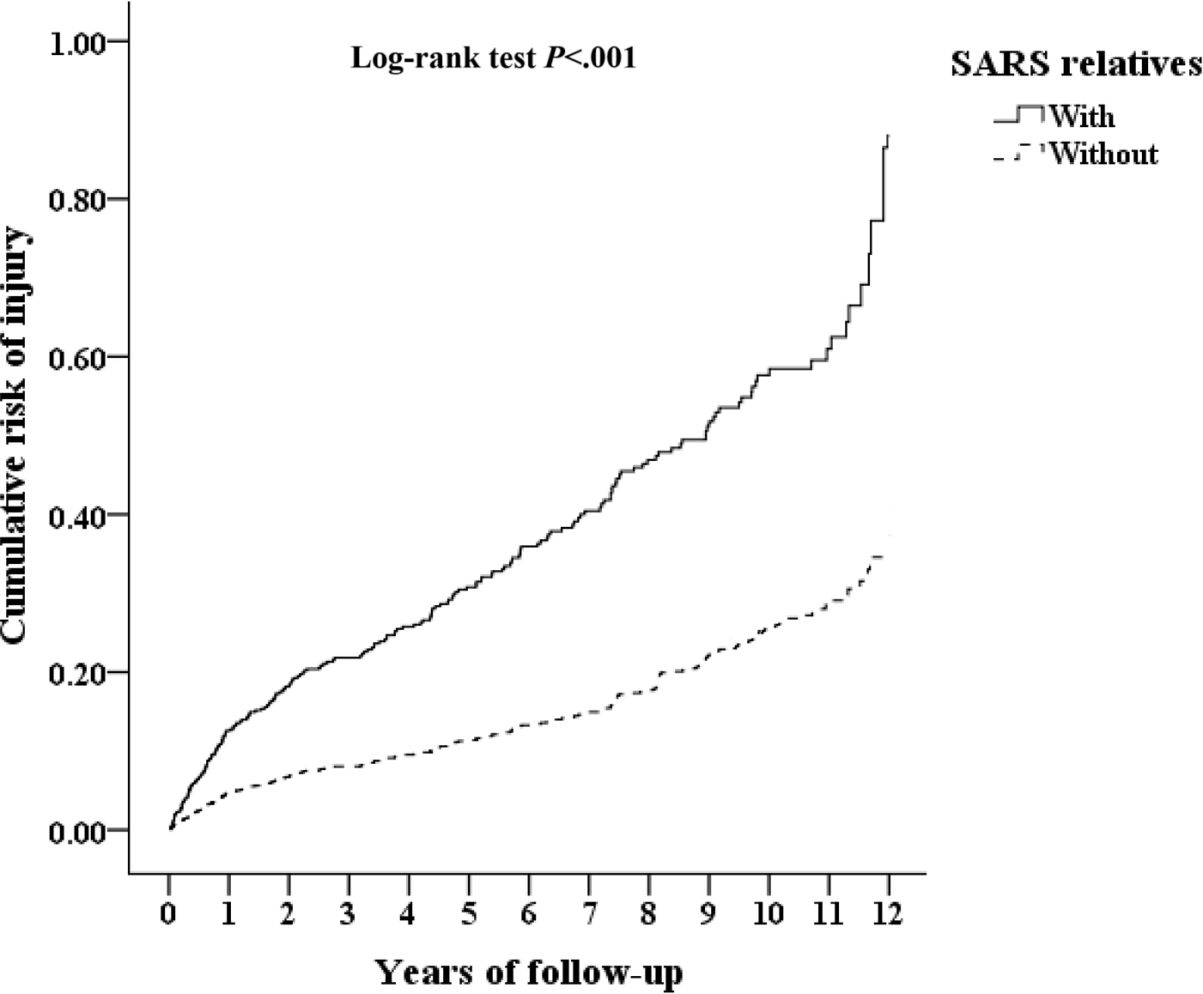
Kaplan-Meier survival curves demonstrating the cumulative incidence of injury among first-degree relatives of survivors of severe acute respiratory syndrome (SARS) compared to matched controls aged 18 years or older in a nationwide retrospective cohort study (Taiwan, 2000‐2015). Statistical significance was evaluated by the log-rank test (*P*<.001).

In total, 699 were relatives of patients with SARS during the study period. During the follow-up period, 187 in the relatives of patients with SARS group (n=699) and 1240 in the control group (n=6990) developed injury (3044.57 vs 2236.71 per 100,000 person-years). Figure 3 reveals that the difference between the two cohorts in the injury was significant (log-rank test; *P*<.001).

### Years From Patients With SARS and Their Relatives to Injuries

The mean time from the index date to the diagnosis of injuries after diagnosis was 3.60 (SD 3.45) years. The mean years to developed injuries in patients with SARS were 3.47 (SD 3.02) years, which was earlier than in patients without SARS (3.61, SD 3.49 years; Table S2 in Multimedia Appendix 1). The mean time from the index date to the diagnosis of injuries after diagnosis was 3.61 (SD 3.45) years. The mean years to the developed injuries in relatives of patients with SARS were 3.54 (SD 3.07) years, which was earlier than relatives of patients without SARS (3.62, SD 3.49 years; Table S2 in Multimedia Appendix 1).

### Analysis of Injury in Patients With SARS and Their Relatives

Table 2 shows the factors of SARS and relatives of patients with SARS, using Fine and Gray’s survival analysis, of the factors associated with the risk of injury. In patients with SARS, the crude AHR was 1.684 (95% CI 1.197‐2.001; *P*<.001), and after adjusting for gender, age, education, monthly insured premiums, urbanization levels, regions of residence, comorbidities, and levels of medical care, the adjusted AHR was 1.631 (95% CI 1.184‐2.011; *P*<.001) for injury. In relatives of patients with SARS, the crude AHR was 1.670 (95% CI 1.283‐2.025; *P*<.001), and after adjusting for gender, age, education, monthly insured premiums, urbanization levels, regions of residence, comorbidities, and levels of medical care, the adjusted AHR was 1.572 (95% CI 1.148‐1.927; *P*<.001) for injury. Factors associated with an increased risk of injury among patients with SARS and their relatives included being male, aged 45–64 years or older than 65 years, having a CCI score of ≥2, residing in urbanization levels 1 and 2, and receiving care from medical centers and regional hospitals.

**Table 2.** AHRs^a^ for factors associated with injury risk among survivors of severe acute respiratory syndrome (SARS) and their first-degree relatives compared to matched controls using Fine and Gray’s competing risk model in a nationwide retrospective cohort study (Taiwan, 2000‐2015)^b^.

	Patients with SARS								Relatives of patients with SARS							
	Competing risk in the model								Competing risk in the model							
	Crude HR^c^	95% CI		*P* value	Adjusted HR	95% CI		*P* value	Crude HR	95% CI		*P* value	Adjusted HR	95% CI		*P* value
SARS (reference: without SARS)	1.684	1.197-2.001		<.001	1.631	1.184-2.011		<.001	1.670	1.283-2.025		<.001	1.572	1.148-1.927		<.001
Male (reference: female)	1.454	1.396-1.572		<.001	1.386	1.267-1.498		<.001	1.428	1.371-1.555		<.001	1.352	1.211-1.409		<.001
Age (reference: 18-44 years)																
45-64 years	1.428	1.199-1.426		<.001	1.401	1.184-1.382		<.001	1.411	1.201-1.529		<.001	1.382	1.10-1.486		<.001
≥65 years	1.677	1.503-1.786		<.001	1.623	1.490-1.777		<.001	1.637	1.512-1.803		<.001	1.597	1.482-1.770		<.001
CCI^d^ (reference: CCI=0)																
1	1.325	1.234-1.496		<.001	1.312	1.204-1.452		<.001	1.311	1.201-1.489		<.001	1.297	1.184-1.446		<.001
≥2	1.620	1.591-1.701		<.001	1.607	1.558-1.693		<.001	1.568	1.401-1.698		<.001	1.525	1.372-1.687		<.001
Urbanization level (reference level: 4)																
1 (the highest)	1.397	1.222-1.489		<.001	1.328	1.201-1.412		<.001	1.375	1.273-1.586		<.001	1.303	1.229-1.512		<.001
2	1.286	1.137-1.402		<.001	1.246	1.101-1.354		<.001	1.312	1.146-1.503		<.001	1.289	1.111-1.487		<.001
Level of care (reference: local hospital)																
Hospital center	1.979	1.733-2.079		<.001	1.928	1.701-2.016		<.001	1.889	1.684-2.011		<.001	1.813	1.607-1.978		<.001
Regional hospital	1.633	1.401-1.976		<.001	1.604	1.389-1.921		<.001	1.606	1.375-1.970		<.001	1.597	1.342-1.784		<.001

a AHR: adjusted hazard ratio.

b This table reports the results of multivariable Cox regression analysis using Fine and Gray’s competing risk model to evaluate the factors influencing injury risk among survivors of SARS (n=285) and their first-degree relatives (n=699), compared to matched non-SARS controls. Hazard ratios and 95% CIs are presented for each factor, including SARS exposure, gender, age, comorbidity index, urbanization level, and hospital level of care. The analysis adjusts for baseline characteristics such as demographic variables, comorbidity burden, and access to health care. Data were collected from the National Health Insurance Research Database during the period 2000‐2015, with statistical significance set at *P*<.05.

c HR: hazard ratio.

d CCI: Charlson Comorbidity Index.

### Subgroup Analysis of Unintentional Injuries and Intentional Injuries in the SARS Cohort and Controls

Table S3 in Multimedia Appendix 1 shows that the SARS cohort had an overall AHR of 1.631 (95% CI 1.184‐2.011; *P*<.001). The AHRs for these unintentional injuries were: overall unintentional injuries 1.711 (95% CI 1.204‐2.112; *P*<.001), poisoning 2.701 (95% CI 1.956‐4.084; *P*<.001), and falls 1.524 (95% CI 1.102‐1.878; *P*=.003). For intentional injuries, the overall AHR was 2.232 (95% CI 1.695-2.879; *P*<.001), with suicide at 2.685 (95% CI 1.947-3.313; *P*<.001) and homicide or abuse at 1.846 (95% CI 1.341-2.277; *P*<.001).

### Subgroup Analysis of Unintentional Injuries and Intentional Injuries in the Relatives of Patients With SARS Cohort and Controls

Table S4 in Multimedia Appendix 1 presents the AHR for the relatives of patients with SARS cohort, which exhibited a significantly increased overall injury risk with an AHR of 1.572 (95% CI 1.148-1.927; *P*<.001). In the category of unintentional injuries, the AHRs were notably elevated: overall unintentional injuries had an AHR of 1.742 (95% CI 1.270-2.135; *P*<.001); traffic-related injuries, 2.003 (95% CI 1.462-2.459; *P*<.001); poisoning, 1.531 (95% CI 1.120‐1.886; *P*<.001); falls, 1.802 (95% CI 1.324‐2.214; *P*<.001); and crushing injuries, 2.469 (95% CI 1.803‐3.026; *P*<.001). Similarly, intentional injuries demonstrated significantly higher AHR: overall intentional injuries, 2.876 (95% CI 2.101‐3.529; *P*<.001); suicide, 2.197 (95% CI 1.603‐2.678; *P*<.001); and homicide or abuse, 4.163 (95% CI 3.032‐5.010; *P*<.001).

### Sensitivity Test for Analysis of the Risk of Injury in Patients With SARS and Their Relatives

Patients with SARS and their relatives were associated with an increased risk of injury compared to the control group. For patients with SARS, the AHR for these injuries was 1.631 (95% CI 1.184‐2.011; *P*<.001). For relatives of patients with SARS, the AHR was 1.572 (95% CI 1.148‐1.927; *P*<.001). Sensitivity analyses revealed that the associations between injury and both the patients with SARS and their relative cohorts remained significant after excluding individuals diagnosed with injury within the first year, as well as when those diagnosed within the first 5 years were excluded (Table 3).

**Table 3.** Sensitivity analysis of injury risk excluding early injury events in survivors of severe acute respiratory syndrome (SARS) and their first-degree relatives compared to matched controls using Fine and Gray’s competing risk model in a nationwide retrospective cohort study (Taiwan, 2000‐2015)^a^.

	Patients with SARS										Relatives of patients with SARS									
	With			Without (reference)			Competing risk in the model				With			Without (reference)			Competing risk in the model			
Sensitivity test	Events	PYs^b^	Rate (per 10^5^ PYs)	Events	PYs	Rate (per 10^5^ PYs)	AHR^c^	95% CI		*P* value	Events	PYs	Rate (per 10^5^ PYs)	Events	PYs	Rate (per 10^5^ PYs)	AHR	95% CI		*P* value
Overall	112	2014.25	5560.38	507	22,191.77	2284.63	1.631	1.184-2.011		<.001	187	6142.09	3044.57	1240	55,438.57	2236.71	1.572	1.148-1.927		<.001
In the first year excluded	101	1812.80	5571.49	483	19,972.30	2418.35	1.618	1.177-2.000		<.001	167	5527.81	3021.09	1189	49,894.25	2383.04	1.529	1.124-1.906		<.001
In the first 5 years excluded	62	1173.25	5284.47	303	12,945.33	2340.61	1.429	1.051-1.867		.001	108	3582.70	3014.49	803	32,339.18	2483.06	1.498	1.095-1.873		<.001

a This table displays results of sensitivity analyses evaluating the robustness of injury risk estimates among survivors of SARS and their relatives by excluding injury events occurring within the first year and first 5 years of follow-up. Person-years, event rates per 100,000 person-years, and adjusted hazard ratios with 95% CIs are reported. Data were derived from the National Health Insurance Research Database in Taiwan and analyzed using Fine and Gray’s model, adjusting for relevant baseline characteristics.

b PY: person-year.

c AHR: adjusted hazard ratio.

## Discussion

### Summary of Main Findings

Our study conducted long-term follow-ups of patients with SARS and their relatives. This longitudinal approach provides a comprehensive understanding of the enduring impacts of SARS on both patients and their families. A systematic review conducted in Singapore observed significant decreases in elective, traumatic, and outpatient orthopedic surgeries by 80%, 47%, and 63%, respectively, since the onset of the pandemic. This review found an overall proportional increase in domestic injuries and polytraumas, which was attributed to patients with minor injuries potentially avoiding treatment to minimize their risk of COVID-19 exposure [50]. Additionally, a study from Michigan reported an overall 45% reduction in orthopedic trauma encounters at a level II trauma center following the implementation of a shelter-in-place order [51]. Other research has similarly demonstrated a trend of decreased pediatric and adult trauma and orthopedic admissions since the beginning of the COVID-19 pandemic. However, none of these studies have specifically examined the incidence of injuries or changes in trauma admissions in the northwest Ohio region [52-58].

In this study, we identified several noteworthy findings regarding injury risks within the SARS cohort and their relatives. Specifically, the SARS cohort exhibited a 1.631-fold increased risk of overall injuries compared to the control group. Delving into the causes of these injuries, unintentional injuries presented a 1.711-fold increase, poisoning incidents rose by 2.701-fold, and falls were associated with a 1.524-fold higher risk. Additionally, the cohort of relatives of patients with SARS demonstrated a 1.572-fold elevated risk of overall injuries. Breaking down the injury causes within this group, unintentional injuries showed a 1.742-fold increase, traffic-related injuries doubled with a 2.003-fold rise, poisoning incidents were up by 1.531-fold, medical-related injuries increased by 1.362-fold, falls were 1.802-fold more likely, and crushing injuries saw the highest increase at 2.469-fold. In this study, “relatives” are defined as first-degree family members (spouses, children, parents, or siblings) sharing the same NHI household registration [7]. Because the NHIRD does not capture cohabitation or caregiving roles, we cannot determine whether these relatives lived with or provided direct care to the patients with SARS. Subgroup analysis (Table S4 in Multimedia Appendix 1) shows that relatives of deceased patients with SARS had an AHR of 2.34 (95% CI 2.10‐2.61), compared to 1.78 (95% CI 1.56‐2.03) for relatives of surviving patients. The higher risk among bereaved relatives may reflect grief-related trauma and family disruption, though increased health care interactions or reporting bias cannot be excluded. Future research should incorporate data on household residency and caregiving responsibilities—such as via linkage to household registries or targeted surveys—to clarify the pathways by which SARS exposure affects relatives’ injury risk.

### Detailed Discussion and Interpretation

Interestingly, in other studies, we have found that falls encounters increased during the COVID-19 pandemic. The impact of COVID-19 on the older population included not only detrimental health effects but also societal effects, such as increased isolation leading to higher rates of depression, anxiety, and dementia [5]. We hypothesize that the incidence of falls increased among the older population due to the decreased availability of professional caregivers during COVID-19. With fewer family members and professional help available, older patients may have been more susceptible to falling. Additionally, in this study, the risk of falls remained elevated 15 years after tracking patients with SARS, which we attribute to the physical weakness resulting from the illness. In contrast, among relatives of patients with SARS, the risk of falls did not increase. These findings highlight the long-term physical vulnerabilities in patients with SARS and suggest that the broader societal disruptions caused by pandemics can have lasting impacts on injury risks within vulnerable populations. The literature indicates that fatigue was the most common symptom among survivors of SARS 18 years after discharge, with osteoporosis and femoral head necrosis being the primary sequelae. The respiratory and hip function scores of survivors of SARS were significantly lower compared to those of the control group. Physical and social functioning at 18 years postdischarge showed improvement compared to that at 12 years but still remained inferior to the controls. Emotional and mental health were fully recovered. Lung lesions detected by computed tomography scans remained consistent at 18 years, particularly in the right upper lobe and left lower lobe. Plasma multiomics analysis indicated abnormal metabolism of amino acids and lipids, an enhanced host immune defense response to bacteria and external stimuli, B-cell activation, and increased cytotoxicity of CD8+ T cells, while showing impaired antigen presentation capacity of CD4+ T cells [6,59-61]. These factors may contribute to the increased risk of falls among patients with SARS.

The persistence of suicide and homicide, or abuse risk morbidities among survivors of SARS and their relatives who participated in this study was alarming. This study identified a particularly noteworthy finding: among patients with SARS, the risk of homicide or abuse was elevated by a factor of 1.846. However, this risk was significantly higher among the relatives of patients with SARS, increasing to 4.163 times. Reviewing results from previous post-SARS cohorts, psychiatric morbidities measured by standardized questionnaires ranged from 10% to 35% in the acute phase of the infection (from the acute phase to 1 month after SARS) [62,63], increasing to 64% at the 1-year follow-up [64]. In the context of the COVID-19 pandemic, research has demonstrated a significant change in the number of nonaccidental trauma encounters, with more cases occurring during the pre-COVID-19 period than during COVID-19 among both adult (15 vs 11 cases) and pediatric (29 vs 9 cases) populations. There is substantial concern that individuals may have been subjected to increased neglect and abuse due to the heightened time spent at home during COVID-19 [8]. Regarding the observed decrease in nonaccidental trauma encounters during COVID-19, concerns have been raised about whether this reduction was due to fewer patients seeking treatment for these traumas rather than a true decrease in the incidence of such events. During the pandemic, time spent engaging in activities outside the home—where nonaccidental trauma could be identified and reported—considerably decreased. In addition to the psychosocial stressors associated with SARS and COVID-19, the long-term adverse health outcomes experienced by survivors of SARS may also serve as a risk factor for accidental injuries among their relatives [65,66]. The rise in suicide rates following the SARS outbreak was a critical public health concern. Previous studies have explored the potential increased risk of suicide among older populations [3,4] and emergency department visitors [67] during and after the SARS outbreak. To our knowledge, this study represents the first investigation into suicide specifically among survivors of SARS. As previously discussed, the association between SARS and suicide risk was found to be statistically significant, even after excluding suicides occurring within the first year postinfection. However, this association was not significant after excluding suicides that occurred within the first 5 years. These findings highlight the importance of vigilant assessment of suicide risk in the years following SARS infection.

To elucidate pathways linking SARS exposure to elevated injury risks, we propose a 3-fold framework. Biological: chronic fatigue, pulmonary fibrosis, and immune dysregulation in survivors of SARS may impair physical resilience and coordination, contributing to a higher fall risk (AHR 1.524, 95% CI 1.102‐1.878; Table S3 in Multimedia Appendix 1) and paralleling neurological sequela observed in survivors of COVID-19 [68]. Psychological: quarantine stress, fear of morbidity or mortality, and economic hardship exacerbate mental-health complications, elevating self-harm risk (suicide AHR 2.685, 95% CI 1.947‐3.313; Table S3 in Multimedia Appendix 1) and accidental injuries. Social: reduced social support and disrupted routines further heighten vulnerability, particularly among isolated individuals. Preventive interventions: regular physical activity has demonstrated efficacy in preventing postinfection mental-health complications [69], suggesting a modifiable strategy to mitigate injury risk in both SARS and COVID-19 populations [70,71].

Factors contributing to this increased burden may include stress from quarantine and isolation, fear of uncertainty and death, impaired physical health following severe viral infection, the economic consequences of SARS, and even adverse emotional responses to SARS-related information [71-73]. These factors collectively underscore the necessity for careful monitoring and proactive intervention to mitigate psychiatric risks in affected populations.

### Limitations

First, although we achieved an excellent balance on observed covariates—gender, age, insurance premium, CCI, geographic location, level of care, and index date (all *P*>.05; Table 1)—our retrospective matched-cohort design cannot definitively establish causality. Unmeasured confounders—such as occupational exposures (eg, health care workers at hospitals and airport or transit staffs) or underlying socioeconomic and environmental factors—may bias our estimates. Indeed, the AHR for homicide or abuse among relatives (AHR 4.163; 95% CI 3.032‐5.010) substantially exceeds that for patients (AHR 1.846; 95% CI 1.341‐2.277; Table S4 in Multimedia Appendix 1), highlighting potential residual confounding. Moreover, our analysis is based exclusively on Taiwan’s 2003 SARS outbreak, which featured distinctive health care responses, social-distancing policies, and cultural norms; therefore, generalizing to other populations or to the COVID-19 pandemic—despite emerging parallels in postinfection neurological sequela—requires caution [68].

Second, the NHIRD does not capture validated injury severity measures (eg, standardized scales), nor does it include laboratory or imaging results or confirmatory diagnostic-test data, limiting our capacity to explore underlying injury mechanisms or to stratify outcomes by clinical severity [40].

Third, patients treated for SARS may have been more health-conscious, potentially leading to an overestimation of injury incidence. Additionally, clinicians may have exercised heightened vigilance in managing survivors of SARS, possibly increasing inpatient referrals. However, sensitivity analyses excluding injuries occurring within the first year and first 5 years after SARS still showed significantly elevated risks (AHR 1.618, 95% CI 1.177‐2.000; AHR 1.429, 95% CI 1.051‐1.867). This suggests that differential treatment decisions alone are unlikely to explain the higher hazard ratios observed.

Finally, our 15-year follow-up spanned periods of major shifts in health care delivery (eg, rapid adoption of telemedicine and reorganization of hospital services for infection control), social-support structures (eg, community quarantine measures and establishment of outreach networks), and injury-reporting workflows (eg, revised triage protocols and updated insurance-claim coding). Such changes likely affected both care-seeking behaviors and the administrative capture of injuries; for instance, outpatient visits decreased by approximately 45% during the SARS outbreak [74], indicating potential underascertainment of minor injuries. Similar patterns of health care avoidance have been observed during the COVID-19 pandemic, highlighting the importance of contextualizing our results within evolving health care and reporting environments.

Future research should include more detailed clinical data, address potential confounders, and consider expanding the cohort to include family members and health care workers for a more comprehensive understanding.

### Conclusions

This study found that survivors of SARS and their relatives had a significantly increased risk of both unintentional and intentional injuries, persisting over 15 years. Relatives faced a heightened risk of homicide or abuse, while survivors showed increased risks of poisoning, falls, and suicide. These findings highlight the need for clinicians to closely monitor the psychological and injury risks in survivors of SARS and their relatives, especially during future outbreaks like COVID-19, to ensure adequate mental health and safety support.

## Supplementary material

10.2196/70608 Multimedia Appendix 1 Supplementary tables.

## Abbreviations

AHR: adjusted hazard ratio
CCI: Charlson Comorbidity Index
*ICD-9-CM*: *International Classification of Diseases, Ninth Revision, Clinical Modification*
NHI: National Health Insurance
NHIRD: National Health Insurance Research Database
SARS: severe acute respiratory syndrome
