# Supplementary material for: COVID-19–Relevant Insights Into the Elevated Risk of Accidental Injuries in Survivors of SARS and Their Relatives in Taiwan: Retrospective Cohort Study
Source: JMIR Public Health Surveill. 2025 Jul 8;11:e70608. doi: 10.2196/70608 (PMC12262147; doi:10.2196/70608)
Supplement: Multimedia Appendix 1 [file publichealth-v11-e70608-s001.docx]

**Table S1.** ICD-9-CM Codes and Definitions for Injury Types Analyzed Among SARS Survivors, Their First-degree Relatives, and Matched Controls in a Nationwide Retrospective Cohort Study (Taiwan, 2000–2015)

|  |  | **ICD-9-CM / definition** |
| --- | --- | --- |
| **Stduy population:**Severe acute respiratory syndrome | SARS | Primary diagnosis was 480.8-480.9 aged 0.75-91 years in ER and IPD between Feb 25^th^ through Jun 15^th^, 2013 |
| **Excluding:**Pneumonia and influenza |  | 480-488 |
| **Events:** Injury |  | 800-999 |
| Unitentional injuries |  | E800-E949 |
| Traffic |  | E800-E849 |
| Poisoning |  | E850-E869 |
| Medical-related |  | E870-E879 |
| Falls |  | E880-E889 |
| Burns and fires |  | E890-E899 |
| Drowning |  | E910 |
| Suffocation |  | E911-E915 |
| Crushing |  | E916-E920 |
| Adverse drug reaction |  | E930-E949 |
| Other unitentional injuries |  | E900-E909, E921-E929 |
| Itentional injuries |  | E950-E979, E990-E999 |
| Suicide |  | E950-E959 |
| Homocide / Abuse |  | E960-E969 |
| Other itentional injuries |  | E970-E979, E990-E999 |
| Itentional unkonwn |  | E980-E989 |
| Without E-Code |  |  |
| **Comorbidities:** CC revised | CCI_R | CCI removed pneumonia and influenza |
| **Medical visits** |  |  |
| Outpatient department | OPD |  |
| Emergency room | ER |  |
| Inpatient department | IPD |  |
| This table provides a comprehensive listing of ICD-9-CM codes and their corresponding definitions for diseases and injury types. The study examines SARS cases diagnosed between February 25 and June 15, 2003, and categorizes injury outcomes (including traffic accidents, poisonings, falls, and intentional injuries) in a nationwide cohort. This dataset spans from 2000 to 2015, using hospitalization records extracted from the National Health Insurance Research Database (NHIRD). The classification of SARS cases and injuries was conducted to assess long-term health outcomes among survivors and their first-degree relatives, excluding pneumonia and influenza from the comorbidity analysis. | | |

**Table S2.** Time Interval from Index Event to First Documented Injury Event in SARS Survivors, Their First-degree Relatives, and Matched Controls in a Nationwide Retrospective Cohort Study (Taiwan, 2000–2015)

| **SARS** | | | | | **SARS relatives** | | | |
| --- | --- | --- | --- | --- | --- | --- | --- | --- |
|  | **Min** | **Median** | **Max** | **Mean ± SD** | **Min** | **Median** | **Max** | **Mean ± SD** |
| With | 0.01 | 2.22 | 11.74 | 3.47 ± 3.02 | 0.01 | 2.76 | 11.79 | 3.54 ± 3.07 |
| Without | 0.01 | 2.65 | 11.88 | 3.61 ± 3.49 | 0.01 | 3.01 | 11.86 | 3.62 ± 3.49 |
| Total | 0.01 | 2.30 | 11.88 | 3.60 ± 3.45 | 0.01 | 2.88 | 11.86 | 3.61 ± 3.45 |
| This table reports the descriptive statistics (minimum, median, maximum, and mean ± standard deviation) for the time intervals (in years) from the index date (SARS diagnosis for patients or matched entry date for controls) to the occurrence of the first documented injury. The data reflects a retrospective analysis conducted between 2000 and 2015. Comparisons are made between SARS survivors, their first-degree relatives, and matched controls, to evaluate the potential effect of SARS exposure on injury risk and latency. This study controls for age, sex, and socioeconomic status. | | | | | | | | |

**Table S3.** Adjusted Hazard Ratios for Injury Subtypes Among SARS Survivors Compared to Matched Controls Using Fine and Gray’s Competing Risk Model in a Nationwide Retrospective Cohort Study (Taiwan, 2000–2015)

| **SARS** | **With** | | | **Without** *(Reference)* | | | **Competing risk in the model** | | | |
| --- | --- | --- | --- | --- | --- | --- | --- | --- | --- | --- |
| **Cause of injury** | **Events** | **PYs** | **Rate (per 10^5^ PYs)** | **Events** | **PYs** | **Rate (per 10^5^ PYs)** | **Adjusted HR** | **95% CI** | **95% CI** | ***P*** |
| Overall | 112 | 2,014.25 | 5,560.38 | 507 | 22,191.77 | 2,284.63 | 1.631 | 1.184 | 2.011 | <0.01 |
| Unitentional injuries | 61 | 2,014.25 | 3,028.42 | 262 | 22,191.77 | 1,180.62 | 1.711 | 1.204 | 2.112 | <0.01 |
| Traffic | 14 | 2,014.25 | 695.05 | 78 | 22,191.77 | 351.48 | 0.000 | 0.965 | 1.659 | 0.064 |
| Poisoning | 22 | 2,014.25 | 1,092.22 | 30 | 22,191.77 | 135.19 | 2.701 | 1.956 | 4.084 | <0.01 |
| Medical-related | 3 | 2,014.25 | 148.94 | 23 | 22,191.77 | 103.64 | 0.963 | 0.689 | 1.194 | 0.33 |
| Falls | 13 | 2,014.25 | 645.40 | 63 | 22,191.77 | 283.89 | 1.524 | 1.102 | 1.878 | 0.03 |
| Burns and fires | 0 | 2,014.25 | 0.00 | 3 | 22,191.77 | 13.52 | 0.000 | - | - | - |
| Drowning | 0 | 2,014.25 | 0.00 | 1 | 22,191.77 | 4.51 | 0.000 | - | - | - |
| Suffocation | 0 | 2,014.25 | 0.00 | 9 | 22,191.77 | 40.56 | 0.000 | - | - | - |
| Crushing | 3 | 2,014.25 | 148.94 | 19 | 22,191.77 | 85.62 | 1.116 | 0.845 | 1.439 | 0.16 |
| Adverse drug reaction | 1 | 2,014.25 | 49.65 | 8 | 22,191.77 | 36.05 | 0.000 | 0.662 | 1.140 | 0.35 |
| Other unitentional injuries | 5 | 2,014.25 | 248.23 | 28 | 22,191.77 | 126.17 | 1.318 | 0.952 | 1.626 | 0.08 |
| Itentional injuries | 6 | 2,014.25 | 297.88 | 19 | 22,191.77 | 85.62 | 2.232 | 1.695 | 2.879 | <0.01 |
| Suicide | 4 | 2,014.25 | 198.59 | 11 | 22,191.77 | 49.57 | 2.685 | 1.947 | 3.313 | <0.01 |
| Homocide / Abuse | 2 | 2,014.25 | 99.29 | 8 | 22,191.77 | 36.05 | 1.846 | 1.341 | 2.277 | <0.01 |
| Itentional unkonwn | 3 | 2,014.25 | 148.94 | 18 | 22,191.77 | 81.11 | 1.230 | 0.892 | 1.515 | 0.12 |
| Without E-Code | 42 | 2,014.25 | 2,085.14 | 208 | 22,191.77 | 937.28 | 1.491 | 1.082 | 1.838 | 0.05 |
| **PYs = Person-years; Adjusted HR = Adjusted Hazard ratio: Adjusted for the variables listed in baseline table.; CI = confidence interval**  This table presents the adjusted hazard ratios (AHR) and 95% confidence intervals (CI) derived from multivariable Cox proportional hazards models using Fine & Gray’s competing risk methodology, accounting for mortality as a competing event. The analysis covers a cohort of SARS survivors and matched controls over the period from 2000 to 2015. Key findings include significantly increased risks for poisoning (AHR = 2.701, 95% CI: 1.956–4.084, p < 0.001), falls (AHR = 1.524, 95% CI: 1.102–1.878, p = 0.003), and suicide (AHR = 2.685, 95% CI: 1.947–3.313, p < 0.001). The model controls for baseline clinical, demographic, and socioeconomic factors, ensuring robust statistical analysis of injury risks. | | | | | | | | | | |

**Table S4.** Adjusted Hazard Ratios for Injury Subtypes Among First-degree Relatives of SARS Survivors Compared to Matched Controls Using Fine and Gray’s Competing Risk Model in a Nationwide Retrospective Cohort Study (Taiwan, 2000–2015)

| **SARS relatives** | **With** | | | **Without** *(Reference)* | | | **Competing risk in the model** | | | |
| --- | --- | --- | --- | --- | --- | --- | --- | --- | --- | --- |
| **Cause of injury** | **Events** | **PYs** | **Rate (per 10^5^ PYs)** | **Events** | **PYs** | **Rate (per 10^5^ PYs)** | **Adjusted HR** | **95% CI** | **95% CI** | ***P*** |
| Overall | 187 | 6,142.09 | 3,044.57 | 1,240 | 55,438.57 | 2,236.71 | 1.572 | 1.148 | 1.927 | <0.01 |
| Unitentional injuries | 88 | 6,142.09 | 1,432.74 | 527 | 55,438.57 | 950.60 | 1.742 | 1.270 | 2.135 | <0.01 |
| Traffic | 30 | 6,142.09 | 488.43 | 156 | 55,438.57 | 281.39 | 2.003 | 1.462 | 2.459 | <0.01 |
| Poisoning | 9 | 6,142.09 | 146.53 | 61 | 55,438.57 | 110.03 | 1.531 | 1.120 | 1.886 | <0.01 |
| Medical-related | 6 | 6,142.09 | 97.69 | 46 | 55,438.57 | 82.97 | 1.362 | 0.974 | 1.656 | 0.08 |
| Falls | 22 | 6,142.09 | 358.18 | 127 | 55,438.57 | 229.08 | 1.802 | 1.324 | 2.214 | <0.01 |
| Burns and fires | 1 | 6,142.09 | 16.28 | 7 | 55,438.57 | 12.63 | 1.481 | 0.957 | 1.826 | 0.07 |
| Drowning | 0 | 6,142.09 | 0.00 | 3 | 55,438.57 | 5.41 | 0.000 | - | - | 0.98 |
| Suffocation | 2 | 6,142.09 | 32.56 | 17 | 55,438.57 | 30.66 | 1.221 | 0.894 | 1.512 | 0.20 |
| Crushing | 9 | 6,142.09 | 146.53 | 38 | 55,438.57 | 68.54 | 2.469 | 1.803 | 3.026 | <0.01 |
| Adverse drug reaction | 2 | 6,142.09 | 32.56 | 16 | 55,438.57 | 28.86 | 1.303 | 0.951 | 1.587 | 0.17 |
| Other unitentional injuries | 7 | 6,142.09 | 113.97 | 56 | 55,438.57 | 101.01 | 1.305 | 0.957 | 1.593 | 0.19 |
| Itentional injuries | 8 | 6,142.09 | 130.25 | 29 | 55,438.57 | 52.31 | 2.876 | 2.101 | 3.529 | <0.01 |
| Suicide | 4 | 6,142.09 | 65.12 | 19 | 55,438.57 | 34.27 | 2.197 | 1.603 | 2.678 | <0.01 |
| Homocide / Abuse | 4 | 6,142.09 | 65.12 | 10 | 55,438.57 | 18.04 | 4.163 | 3.032 | 5.010 | <0.01 |
| Itentional unkonwn | 6 | 6,142.09 | 97.69 | 45 | 55,438.57 | 81.17 | 1.372 | 0.708 | 1.862 | 0.40 |
| Without E-Code | 85 | 6,142.09 | 1,383.89 | 639 | 55,438.57 | 1,152.63 | 1.364 | 0.677 | 1.850 | 0.43 |
| **PYs = Person-years; Adjusted HR = Adjusted Hazard ratio: Adjusted for the variables listed in baseline table.; CI = confidence interval**  This table displays the AHR and 95% CI for injury subtypes among the first-degree relatives of SARS survivors, using the same statistical approach as in Table S3. The competing risk model accounts for mortality as a competing event and adjusts for age, sex, socioeconomic status, and clinical comorbidities. Significant findings include elevated risks for traffic-related injuries (AHR = 2.003, 95% CI: 1.462–2.459, p < 0.001), falls (AHR = 1.802, 95% CI: 1.324–2.214, p < 0.001), and crushing injuries (AHR = 2.469, 95% CI: 1.803–3.026, p < 0.001) among SARS survivors’ relatives. Data is derived from Taiwan’s NHIRD, spanning from 2000 to 2015, ensuring a comprehensive assessment of injury outcomes among a large cohort. | | | | | | | | | | |
